# Assessing the Accuracy of Bone Resection by Cutting Blocks in Patient-Specific Total Knee Replacements

**DOI:** 10.5402/2012/509750

**Published:** 2012-05-20

**Authors:** Cheng Hong Yeo, A. Jariwala, N. Pourgiezis, A. Pillai

**Affiliations:** ^1^Division of Orthopaedics and Trauma, The Queen Elizabeth Hospital, Adelaide, SA 5011, Australia; ^2^Department of Orthopaedic and Trauma Surgery, Toowoomba Hospital, Toowoomba, QlD, Australia; ^3^Department of Orthopaedic and Trauma Surgery, TORT Centre, Ninewells Hospital and Medical School, University of Dundee, Dundee DD19SY, UK; ^4^The Queen Elizabeth Hospital, Adelaide, SA 5011, Australia; ^5^Department of Orthopaedic and Trauma Surgery, Ninewells Hospital, Ward 18-19, Level 5, Dundee, UK; ^6^The University of Adelaide, Adelaide, SA 5005, Australia

## Abstract

*Introduction*. The key to a successful total knee arthroplasty (TKA) is the restoration of the mechanical axis with balanced flexion and extension gaps. Patient-specific cutting block technique has been the latest development in total knee arthroplasty. This technique uses a magnetic resonance image (MRI) of the patient's symptomatic knee to create bone models and cutting jigs. This study was designed to evaluate the intraoperative accuracy of the patient-specific cutting block as compared to the preoperative template. *Methods*. Visionaire (Smith and Nephew, Genesis 2 Knee Arthroplasty) patient-specific TKA was used in all patients. An independent research officer was responsible for measuring all the resected articular surfaces of femur and tibia during surgery and compared it to the cutting block manufactured according to the preoperative template. Seven different measurements from each patient were obtained; four different measurements from the femur and three from the tibia were recorded. The differences between the actual resections made intraoperatively, as compared to the original pre-operative templates, were noted as the error. The surgical team was blinded to the measurements of the resections and the calculations of the errors. *Results*. Twenty-six Visionaire patient-specific TKA were included in the study. A total of 182 readings of bone resections made intraoperatively (seven for each patient). Eighty five percent of all collected readings were below the error margin of ≤1.5 mm. Size of resection had no effect on the error margin. All patients had satisfactory post-operative alignment, and at discharge all 26 patients achieved more than 90° of knee flexion. *Conclusion*. This observational study provides evidence that patient-specific TKA is comparable to other forms of TKA and may have some distinct advantages. In addition, we have shown that the cutting blocks are able to consistently deliver accurate cuts that are reproducible. We recommend intra-operative measurement of the bone resection and its comparison with the cutting block as a routine surgical step to confirm the MRI scan data, block placement, and instant validation of the bony resection before implant placement.

## 1. Introduction

The key to a successful total knee arthroplasty (TKA) is the restoration of the mechanical axis with balanced flexion and extension gaps. In recent years, rotational malalignment of components has been implicated as a cause of pain after TKR [1]. Navigation TKA was developed to improve the precision of knee implant placement and therefore its longevity. No clear advantage over conventional TKA, difficulties with accurate landmark registration, increased operative time, and associated cost have all been hurdles for navigated TKA to dominate the knee arthroplasty market [2, 3].

Patient-specific cutting block technique (patient-specific TKA) has been the latest development in TKA. This technique uses magnetic resonance imaging (MRI) of the patient's symptomatic knee to create bone models and cutting jigs. This MRI image is used to create three-dimensional models and cutting blocks of the distal femur and proximal tibia. These models are then used with standard TKA operating systems to undertake TKA surgery [4].

The manufacturers and proponents of patient-specific TKA claim that this new technology is able to reproduce exact measurements, possibly has a shorter operating time and is easier to use with shorter learning curve as compared to other types of joint arthroplasty technologies, especially in comparison to navigation. In addition, patient-specific TKA is believed to be more reproducible and cost-effective.

Currently, there are no studies or data available on the accuracy of patient-specific cutting blocks. It would be important to know if the bone resected by the patient-specific blocks actually matches the preoperative sizing. This study was designed to evaluate the intraoperative accuracy of the patient-specific cutting block as compared to the preoperative template.

## 2. Methods

This prospective study was conducted at the Queen Elizabeth Hospital, Adelaide Australia. Patients admitted to the Arthroplasty unit over a period of nine months were included in the study. The inclusion criteria were patients with primary osteoarthritis of knee who were to found suitable to undergo patient-specific TKA. Patients with rheumatoid or secondary osteoarthritis, major deformity or those that needed additional procedures were excluded from the study. All patients had been informed about the study during the preoperative consultation. Ethical approval for the study was not needed as no new intervention or new treatment was initiated or considered.

**Figure 1:**
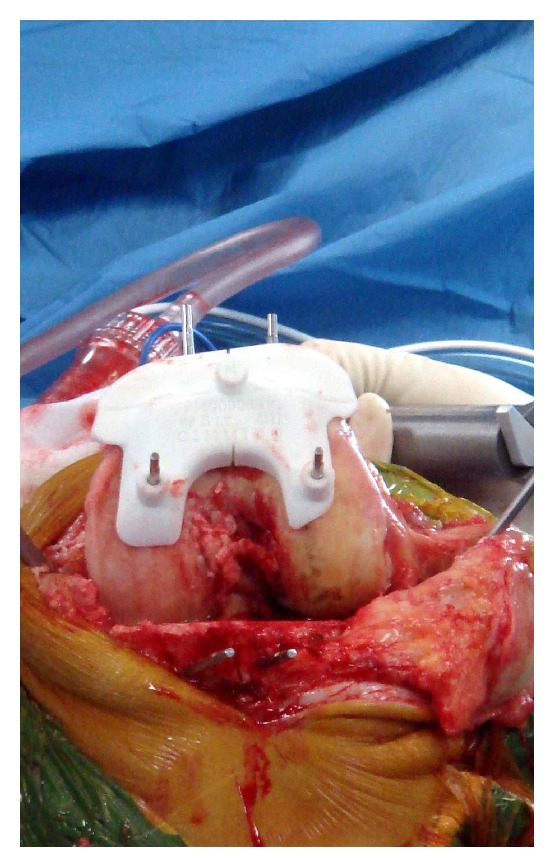
Cutting block positioned over distal femur.

Once patients had been listed for patient-specific TKA they underwent an MRI and long leg weight bearing radiographs. The radiological information was utilised to create virtual model of the patient's knee, and a preoperative plan with approximate sizing and bone cuts was designed. Once verified by the operating surgeon, actual models and cutting blocks of patient's articulating surfaces were created using prototyping technology (Figure 1).

**Figure 2:**
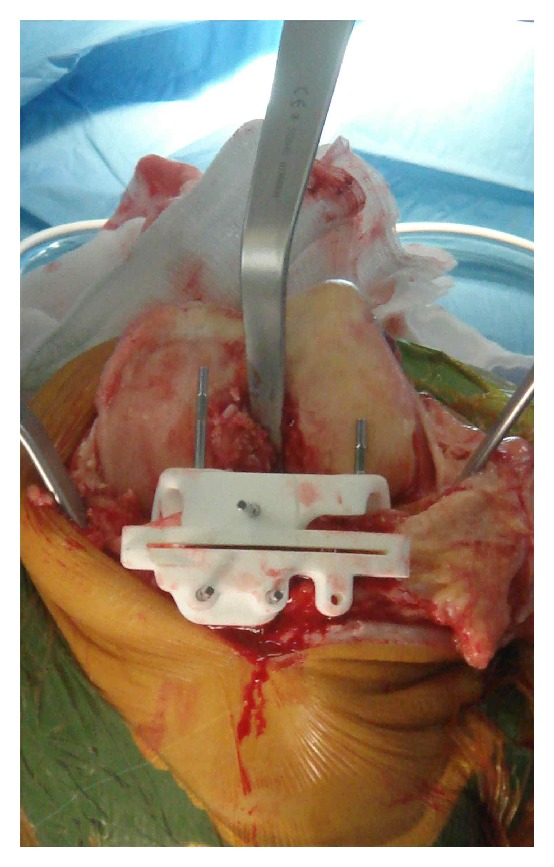
Cutting block positioned over proximal tibia.

Surgery was performed by five orthopaedic specialists and two orthopaedic fellows. All TKAs were performed using a medial parapatellar approach. Visionaire (Smith and Nephew, Genesis 2 Knee Arthroplasty) patient-specific TKA was used in all patients. The cutting block fitted exactly on the articulating surface (Figures 1 and 2). Once the cuts were made, the resected bone was collected by the scrub nurse and handed to an independent research officer, who was responsible for measuring the resected articular surfaces and comparing it to the cutting block. The surgical team was blinded to the measurements and the calculations of the errors. Postoperative clinical and radiological assessments were undertaken by the orthopaedic team.

A micrometer was used to record the cuts. Before each reading was taken, the micrometer was calibrated to zero. The same standard 0.5 micrometer (which could record increments of 0.5 millimetres) was used throughout the study. Three readings for each of the measurements were taken, and an average value was recorded.

Seven different measurements from each patient were recorded. Four different measurements from the femur and three from the tibia were recorded. On the femoral side: distal medial condylar resection (DMC), distal lateral condylar resection (DLC), posterior medial condylar resection (PMC), and posterior lateral condylar resection (PLC) were considered. While on the tibial side: proximal lateral plateau resection (PLP), proximal medial plateau resection (PMP) and resection to the tibial eminence (RE) were noted.

The differences between the actual resections made intraoperatively, as compared to the original preoperative templates, were noted as the error. Errors were categorized into five major groups: 0 mm, 0.5 mm, 1.0 mm, 1.5 mm, 2.0 mm, and ≥2.5 mm.

A saw blade with the thickness of 1.37 mm was used for all TKAs. The thickness was recorded was also measured.

The accuracy of the intra-operative resection (determined by the margin of error recorded) was the primary outcome of the study. In addition, range of motion of knee, the post-operative alignment and post-operative complications such as surgical wound dehiscence, infection, or stiffness were noted as the secondary immediate to short-term outcomes.

## 3. Results

Twenty-six Visionaire patient-specific TKA were included in the study. There were 22 females and four males with a mean age of 70 years with an age range between 49 and 83 years. There were 14 right and 12 left-sided TKAs. Primary osteoarthritis was the diagnosis in all patients. Preoperatively, seven patients had valgus deformity of the operated knee, while 19 had varus deformity. The cutting blocks accurately fitted the distal femur and the proximal tibia with no mismatch. The mean operative duration for patient-specific TKA was 116 minutes. The operating duration was recorded from the time of induction of anaesthesia to the application of dressing. There were no major intra-operative complications or difficulty reported by any of the operating surgeons while using the cutting blocks or its instrumentation.

**Figure 3:**
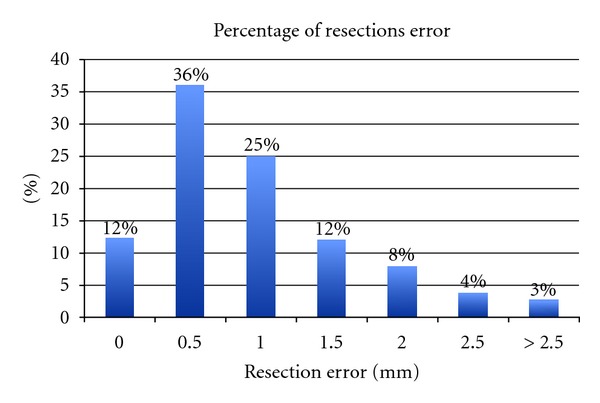
Proportions of errors noted during the study.

A total of 182 readings were collected (seven for each patient). Eighty-five percent of all collected readings were below the error margin of ≤1.5 mm, and 75% of the total readings were actually ≤1 mm more than the original template. There were only 7% of the overall readings that were ≥2.5 mm more than the original preoperative template. Highest error recorded was 3.0 mm (3%), while in 12% of measurements there was no error or deviation from the templated resection (0.0 mm) (Figure 3).

**Figure 4:**
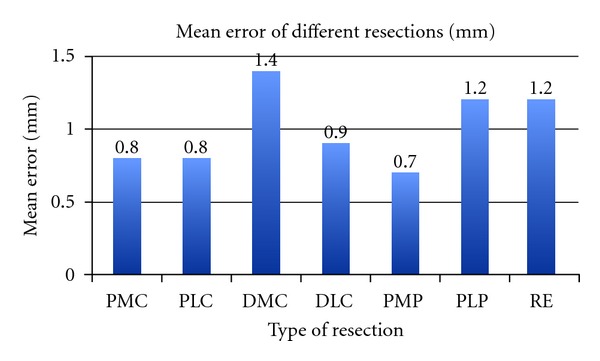
Errors noted for seven different resections.

The distal medial condylar (DMC) resections recorded the highest mean error of all seven resections (1.4 mm). The proximal medial plateau (PMP) resections had the lowest mean error (0.7 mm) (Figure 4). However, in terms of overall bone resection, the femoral subsets were more accurate than the tibial subsets.

**Figure 5:**
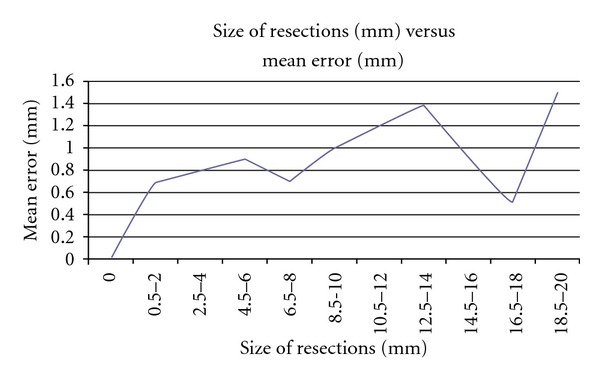
Errors noted for sizes of bone resections.

This study also looked into effects of the sizes of resection made and the error of the resections. However, there were no major differences showed in mean errors of different sizes of resection (Figure 5).

At discharge, all 26 patients achieved more than 90° of knee flexion. All patients had satisfactory post-operative alignment of the TKA on post-operative radiographs both on saggital and coronal views.

Postoperatively, one patient developed deep infection of the right TKA at day six treated with open washout, debridement and change of plastic spacer along with intravenous antibiotics. The patient had a satisfactory outcome at the last followup.

## 4. Discussion

Achieving the correct rotation and alignment of the implant components is critical for the short- and long-term survival of TKA. Evidence from various studies informs us that knee implants malpositioned by more than 3 degrees had a 24% loosening rate at three years after surgery as compared with 3% for correctly aligned implants [1, 5–7].

Patient-specific TKA is the latest addition in the TKA domain [4]. The ability of preoperatively templating the knee with a view to accurately align the knee, deciding upon bone cuts and ability to choose approximate sizes helps the operating surgeons to be better prepared. Additionally, this may also reduce the number of instruments needed to perform TKA thus reducing overall costs and inventory needed.

This study investigated the accuracy of the actual intra-operative resections as compared with the proposed resections.

Comparison between the resected surfaces and the cutting blocks revealed that 85% of the overall readings recorded were within the error margin of 1.5 mm with the highest error being only 3 mm different from the preoperative proposed resection size. It was noted throughout the 9-month study period that the errors were higher in the beginning of the study period as compared to later in the study period. This was accepted as part of the learning curve with the new technology.

All 26 patients achieved more than 90 degrees range of motion at discharge and had satisfactory clinical and radiological outcome. No particular complications were noted from the patient-specific TKA in comparison to those that occur with conventional TKA. There was one infection that had a good outcome with early washout, debridement, and standard antibiotic regime.

Several observations were made by the surgical team, although these were not a part of the research questions. One of the most notable differences reported by the surgeons and by the independent research officer was the reduction in the number of steps required and total time for the patient-specific TKA comparing to the conventional TKA undertaken in the unit. Patient-specific TKA obviates the need for intramedullary or extramedullary alignment guides. In addition, the preoperative template had taken into consideration the presence of the periarticular osteophytes in the preoperative template. Hence, there was no need to resect them separately. We could not objectively comment on these as they were not studied specifically.

Patient-specific TKA proponents recommend its use to improve the accuracy and alignment in TKA. Precise knowledge of patients' articular anatomy, preoperative ability to accurately realign the knee and select appropriate cutting blocks and implant sizes is a distinct advantage offered by patient-specific TKA. As most of the decision making is done pre-operatively, and patient-specific cutting block exactly matches the articular surface, this may reduce the steep learning curve associated with any new TKA. In addition, it proposes to reduce surgical steps, inventory, and possible overall surgical time.

These particular advantages offered by patient-specific TKA coupled with the fact that the surgical results and complications match those of well-established TKA are reassuring. In addition, it would be important to analyse the medium to long-term clinical and radiological results to firmly establish the place of patient-specific TKA.

This observational study provides evidence that patient-specific TKA is comparable to other TKA and may have some distinct advantages. In addition, we have shown that the cutting blocks are able to consistently deliver accurate cuts that are reproducible. We recommend intra-operative measurement of the bone resection and its comparison with the cutting block to give instant validation of the bony resection. In addition, it offers instant validation of the MRI scan data and block placement before implantation of the arthroplasty. This step should be considered as a routine surgical practice for those adopting patient-specific TKA, as block placement is the most crucial step in the procedure, which determines the final implant placement and crucially the final outcome.

## References

[1] Nicoll D, Rowley DI (2010). Internal rotational error of the tibial component is a major cause of pain after total knee replacement. *Journal of Bone and Joint Surgery B*.

[2] Kim YH, Kim JS, Choi Y, Kwon OR (2009). Computer-assisted surgical navigation does not improve the alignment and orientation of the components in total knee arthroplasty. *Journal of Bone and Joint Surgery A*.

[3] Spencer JM, Chauhan SK, Sloan K, Taylor A, Beaver RJ (2007). Computer navigation versus conventional total knee replacement: no difference in functional results at two years. *Journal of Bone and Joint Surgery*.

[4] Lombardi AV, Berend KR, Adams JB (2008). Patient-specific approach in total knee arthroplasty. *Orthopedics*.

[5] Fehring TK, Odum S, Griffin WL, Mason JB, Nadaud M (2001). Early failures in total knee arthroplasty. *Clinical Orthopaedics and Related Research*.

[6] Jeffery RS, Morris RW, Denham RA (1991). Coronal alignment after total knee replacement. *Journal of Bone and Joint Surgery B*.

[7] Ritter MA, Faris PM, Keating EM, Meding JB (1994). Postoperative alignment of total knee replacement: its effect on survival. *Clinical Orthopaedics and Related Research*.

